# Decreased Core-Fucosylation Contributes to Malignancy in Gastric Cancer

**DOI:** 10.1371/journal.pone.0094536

**Published:** 2014-04-14

**Authors:** Yun-Peng Zhao, Xin-Yun Xu, Meng Fang, Hao Wang, Qing You, Chang-Hong Yi, Jun Ji, Xing Gu, Ping-Ting Zhou, Cheng Cheng, Chun-Fang Gao

**Affiliations:** 1 Department of Laboratory Medicine, Eastern Hepatobiliary Surgery Hospital, Second Military Medical University, Shanghai, China; 2 Department of General Surgery, Changzheng Hospital, Second Military Medical University, Shanghai, China; 3 Department of Laboratory Medicine, Changzheng Hospital, Second Military Medical University, Shanghai, China; Duke University Medical Center, United States of America

## Abstract

The object of the study is to identify N-glycan profiling changes associated with gastric cancer and explore the impact of core-fucosylation on biological behaviors of human gastric cancer cells. A total of 244 subjects including gastric cancer, gastric ulcer and healthy control were recruited. N-glycan profiling from serum and total proteins in gastric tissues was analyzed by DNA sequencer-assisted fluorophore-assisted capillary electrophoresis. The abundance of total core-fucosylated residues and the expression of enzymes involved in core-fucosylation were analyzed with lectin blot, quantitative reverse transcription-polymerase chain reaction, western blot, Immunohistochemical staining and lectin-histochemical staining. The recombinant plasmids of GDP-fucose transporter and α-1,6-fucosyltransferase (Fut8) were constructed and transfected into gastric cancer cell lines BGC-823 and SGC-7901. CCK-8 and wound healing assay were used to assess the functional impact of core-fucosylation modulation on cell proliferation and migration. Characteristic serum N-glycan profiles were found in gastric cancer. Compared with the healthy control, a trianntenary structure abundance, peak 9 (NA3Fb), was increased significantly in gastric cancer, while the total abundance of core-fucosylated residues (sumfuc) was decreased. Core-fucosylated structures, peak6(NA2F) and peak7(NA2FB) were deceased in gastric tumor tissues when compared with that in adjacent non-tumor tissues. Consistently, lens culinaris agglutinin (LCA)-binding proteins were decreased significantly in sera of gastric cancer, and protein level of Fut8 was decreased significantly in gastric tumor tissues compared with that in adjacent non-tumor tissues. Upregulation of GDP-Tr and Fut8 could inhibit proliferation, but had no significant influence on migration of BGC-823 and SGC-7901 cells. Core-fucosylation is down regulated in gastric cancer. Upregulation of core-fucosylation could inhibit proliferation of the human gastric cancer cells.

## Introduction

The gastric cancer (GC) is the fourth most common cancer and the second most common cause of cancer related death worldwide [1]–[3], particularly prevalent in many Asian countries, especially China[4]. Protein glycosylation is one of the most common posttranslational modifications made to proteins [5]. Glycans can be attached to proteins either via an amide group (N-linked glycosylation) or a hydroxyl group (O-linked glycosylation), which occur through different biosynthetic pathways and potentially have independent functions [6]. N-linked glycosylation plays fundamental roles in many biological processes such as cell adhesion, cell migration, and signal transduction [7]. Abnormal expression of N-linked glycoproteins has been observed in various diseases [8]–[10]. Upon in-depth characterization of N-linked glycoproteins and disease-associated glycosylation changes, several methodologies have been developed. In our previous study, we have identified some N-glycan markers in heptatocellular carcinoma (HCC) and colon cancer using a capillary based electrophoresis called DNA sequencer-assisted fluorophore-assisted capillary electrophoresis (DSA-FACE) [11]. In addition, it has been reported that α-1, 6-fucosyltransferase (Fut8) activity and expression is increased in several human cancers, suggesting a role for this enzyme in tumor development and progression, such as HCC [12], colorectal cancer [13], nonsmall cell lung cancer [14] and ovarian serous adenocarcinoma [15]. Altered core-fucosylation is one of the most important abnormal glycosylated modification identified in malignancies. Fut8 catalyzes the transfer of fucose from guanosine diphosphate (GDP)-fucose to the innermost GlcNAc of hybrid and complex N-linked oligosaccharides via an α-1,6-linkage, resulting in core-fucosylated glycoproteins [16]–[17] and altering biological function of resulting glycoproteins [18]. Although many studies have reported the association between altered core-fucosylation and other aggressive tumors, to our knowledge, the influence of core-fucosylation on gastric cancer remains unknown. In this study, we analyzed N-glycan profiling with DSA-FACE in both serum samples from gastric cancer, gastric ulcer, healthy controls and tissue proteins from tumors and adjacent non-tumors. Then extend the functional research upon the identified specific glycosylations.

## Materials and Methods

### Ethics Statement

The study protocol was approved by the Chinese Ethics Committee of Human Resources at the Second Military Medical University. All study participants provided written informed consent.

### Study population

In total, 105 patients with gastric cancer (n = 80) and gastric ulcer (n = 25) were enrolled between December 2007 and October 2010 at Changzheng Hospital of the Second Military Medical University (Shanghai, China). All cases with gastric cancer enrolled were histopathologically confirmed by 2 pathologists and cases with gastric ulcer recruited were confirmed by gastroscope. Patients with gastric cancer who received preoperative chemotherapy, and who had other diseases including infections were excluded from the study. Serum samples were obtained before surgical resections. For a control group, 139 age matched, healthy volunteers were enrolled from a pool of cancer-free individuals who visited the same hospital for a regular physical examination and who volunteered to join the research during the same period. We defined a healthy individual as someone who was deemed free of diseases (including no history of cancer) at health check-up. The following clinical characteristics of subjects were obtained at the time of whole blood collection. A summary of the data from these subjects is provided in Table 1. The progression of all patients with gastric cancer was classified according to the Union for International Cancer Control (UICC) TNM staging criteria for gastric cancer, 13 patients (16.25%) had stage I, 24 patients (30.00%) had stage II, 30 patients (37.5%) had stage III, and 13 patients (16.25%) had stage IV. The average age of all patients was 54.35±6.69 including 60 males and 20 females. Serum was collected using a standard protocol from whole blood, treated by centrifugation at 10,000 g for 20 minutes, and stored at −80°C.

**Table 1:** Demographic and clinical characteristics of subjects in research groups.

	research groups (number)		
Characteristic	healthy control	gastric ulcer	gastric cancer
	(means±SD,n = 139)	(means±SD,n = 25)	(means±SD,n = 80)
Age, years	54.82±5.70	54.52±7.33	54.35±6.69
Male (n, %)	79(56.83%)	19(76.00%)	60(75.00%)
ALT (IU/L)	21.49±9.52	20.09±11.41	22.79±15.45
AST (IU/L)	19.67±4.81	21.53±4.24	23.79±12.29
Tbil (µmol/L)	11.93±3.84	12.56±4.59	15.70±7.10
TP (g/dL)	76.03±3.93	73.79±6.49	69.99±8.34
CEA>5 ng/ml n(%)	0	1(4%)	12(15.0%)
CA19-9>35 U/ml n(%)	0	3(12%)	12(15%)
CA724>35 U/ml n(%)	0	1(4%)	6(7.5%)
CA125>35 U/ml n(%)	0	0(0)	9(11.25%)
TNM			
I			13(16.25%)
II			24(30%)
III			30(37.5%)
IV			13(16.25%)

Enumeration data are expressed as n (%);

ALT (alanine aminotransferase), AST (aspartate aminotransferase), Tbil (total bilirubin),

TP (total protein), CEA (carcino-embryonic antigen), CA19-9 (carbohydrate antigen 19-9),

CA724 (carbohydrate antigen 724), CA125 (carbohydrate antigen 125)

### N-Glycan profiling from serum proteins

Serum protein N-glycan analyses were performed as described previously [11]. Briefly, the N-glycans present on the proteins in 2 µl of serum were released with peptide N-glycosidase-F (PNGaseF) (New England Biolabs, Boston, Mass) and then labeled with 8-aminonaphtalene-1, 3, 6-trisulphonic acid (APTS) (Invitrogen, Carlsbad, Calif).Sialic acid was removed with arthrobacter ureafaciens sialidase (Roche Bioscience, Palo Alto, Calif), and the processed samples were analyzed with DSA-FACE technology using a capillary electrophoresis-based ABI3500 Genetic Analyzer (Applied Biosystems, Foster City, Calif). The 9 most intense peaks that were detected in all samples (together, these peaks accounted for >90% of total serum N-glycans) were analyzed using GeneMapper software (Applied Biosystems). Each structure of N-glycan was described numerically by normalizing its height to the sum of the heights of all peaks.

### Tissue samples

All tissues were used in accordance with the Institutional Review Board Regulations of the Second Military Medical University. Tissue samples were obtained from10 of 80 patients with gastric cancer. Tissue samples included 2 slices, a tumor tissue and a adjacent non-tumor tissue. Paired tissue samples (approximately 0.5×0.5×0.5 cm^3^, duplicated for each sample) selected were either immediately frozen at −80°C for RNA and protein extraction or fixed in 10% buffered formalin for up to 24 hours and then processed into a paraffin embedded block and stored at room temperature for histochemical staining.

### N-Glycan profiling from tissue proteins and structural analysis using exoglycosidase digestion

Proteins were extracted from approximately 25 mg frozen tissue specimens. The tissue specimens were suspended and pestled in lysis buffer containing protease inhibitors cocktail (Roche Diagnostics, Meylan, France). Unlysed parts were removed by centrifugation (12000 g for 10 minutes at 4°C) twice. The concentration of solubilized proteins was determined using the BCA kit(Pierce Biotechnology/Thermo Fisher Scientific, Rockford, IL), and the protein samples were stored at −80°C until use. A total of 40 µg tissue proteins were used to establish N-glycan profiles using regular N-Glycan profiling method the same as that performed in serum described above. To identify core-α-1, 6-fucosylated N-glycan structures, an appropriate number of APTS-labeled N-glycans were digested with both arthrobacter ureafaciens sialidase and bovine kidney α-1, 6-fucosidase (Prozyme, San Leandro, CA, USA). DSA-FACE technology was used to analyze the digestion products and GeneMapper software was used to analyze the height of the peaks.

### RNA extraction and quantitative real-time PCR

Total RNAs were extracted from frozen tissues and cells, using total RNA extraction kit according to the manufacturer's instructions (Axygen Biosciences, Hangzhou, China). The purity and concentration of RNA were determined by spectrophotometer (Eppendorf, Hamburg, Germany), and then stored at −80°C until use. The cDNA was synthesized using ReverTra Ace-α-RT-PCR kit (Toyobo Co., Osaka, Japan) according to the manufacturer's instructions. Primers were designed using the Primer Express program (Applied Biosystems; the sequences are shown in Table S1). Quantitative real-time PCR was performed using the SYBR® Green Real-time PCR Master Mix kit (Toyobo Co., Osaka, Japan) and was analyzed on applied Biosystems 7300 Real-Time PCR system (ABI, Foster City, CA, USA). All assays were carried out independently in triplicate. PCR cycling consisted of denaturation at 95°C for 5 minutes, followed by 40 cycles at 95°C for 15 seconds and at 59°C or 55°C for 15 seconds, and detection for 45 seconds at 72°C. The relative amount of Fut8 or guanosine diphosphate (GDP)-fucose transporter (GDP-Tr) transcripts in each sample was normalized to the housekeeping gene β-actin and Glyceraldehyde-3-phosphate dehydrogenase (GAPDH) by subtracting the cycle. The levels of gene expression were determined using Delta-Delta Ct method. Absolute transcript expression values beyond 40 cycles were considered below detectable levels. Melt curves were checked for each reaction to guarantee that a single product was amplified.

### Western blot and Lectin blot

Proteins were extracted from frozen tissues with the procedure described above, and a total of 50 µg proteins were separated by electrophoresis in 10% sodium dodecyl sulfate-polyacrylamide gel (SDS-PAGE). Gels were stained with coomassie blue G250 or the proteins in the gel were transferred to a nitrocellulose membrane () for the detection of Fut8 or the percentage of core-fucosylated proteins. For serum samples, a total of 85 cases from gastric cancer (n = 80), gastric ulcer (n = 25) and healthy control (n = 30) were used for SDS-PAGE and lectin-blot. The membranes were blocked overnight at 4°C with 5% nonfat milk protein or 5% bovine serum albumin in tris-buffered saline [140 mM NaCl, 10 mM Tris-HCl (TBS)] and then incubated for 2 hour at room temperature with 1∶100 diluted anti-human Fut8 antibody (15C6, anti-human Fut8, Fujirebio Corp., Japan) or 5 µg/ml of biotinylated lens culinaris agglutinin A (LCA) (Vector Laboratories, Burlingame, Calif) in TBS containing 0.05% Tween-20 (TBST buffer), and then incubated with a 1∶10,000 dilution of fluorescence-labeled secondary antibodies (LI-COR Biosciences, Lincoln, NE) or IRDye 800CW-streptavidin (LI-COR Biosciences) for 1 hour at room temperature. The membranes were developed with the Odyssey IR Imaging System. Anti-beta actin (1∶2000) was used for internal reference antibody for western-blot. Purified albumin was used as negative control for lectin blot and total protein stained with coomassie blue was used to calculate the percentage of core-fucosylated protein.

### Immunohistochemical (IHC) staining and lectin-histochemical staining

The fixed tissue specimens embedded in paraffin were cut into 4-mm-sick slices for immunohistochemical staining for Fut8 and lectin-histochemical staining for LCA. After undergoing deparaffinization, rehydration, endogenous peroxidase blocking, and antigen retrieval (10 mM Tris/1 mM EDTA, pH 9.0, autoclave treated), specimens were incubated with a mouse monoclonal antibody against human Fut8 (1∶100) or biotinylated LCA (1∶500) overnight at 4°C, and then with horseradish peroxidase-conjugated secondary antibody or Fluorescein labeled streptavidin at 37°C for 30 min. Nuclei were counterstained with hematoxylin or Draq5. Negative control was composed of identically treated histological sections with mouse IgG to replace mouse Fut8.

### Cell lines and culture

The human gastric cancer cell lines, BGC-823 and SGC-7901, were purchased from the Shanghai Institute of Cell Biology, Chinese Academy of Sciences and cultured at 37°C under 5% CO_2_ in RPMI 1640 medium and DMEM medium (Invitrogen Life Technologies, Carlsbad, CA), respectively, with 10% FBS, 1% glutamine, and 1% antibiotic solution.

### Construction of GDP-Tr and Fut8 Recombinant Plasmids and transfection of gastric cancer cells

The pEGFP-N1-GDP-Tr and the pEGFP-N1-Fut8, the plasmid vector encoding human GDP-Tr or Fut8, was generated by inserting GDP-Tr or Fut8 cDNA into a pEGFP-N1 vector (Clontech Laboratories, Inc., Mountain View, CA, USA), containing a CMV promoter and SV40 early promoter, as well as the neomycin/kanamycin resistance gene of Tn5. The vector backbone also contains an SV40 origin of replication in mammalian cells. The multiple cloning site (MCS) is next to the immediate early promoter of CMV. The human GDP-Tr or Fut8 gene was cloned from the mRNA extracted with TRIzol reagent (Invitrogen, Carlsbad, USA) from human liver tissue by performing RT-PCR using the primers (Table S2) in which the Xho I or Kpn I restriction sites were included, digesting with Xho I or Kpn I and ligating into the pEGFP-N1. The pEGFP-N1-GDP-Tr or the pEGFP-N1-Fut8 was transfected into Escherichia coli DH5α for amplification and DNA sequencing was used to ensure the fidelity. Plasmid DNA was prepared using the Qiagen DNA mini kit (Qiagen, Hilden, Germany) as per the manufacturer's instructions. Plasmids were transfected into the human gastric cancer cell lines, BGC-823 and SGC-7901 at 80% confluence and 5×10^5^ cells per well in a six-well plate using the Lipofectamine™ 2000 (Invitrogen, Carlsbad, USA) according to the manufacturer's instructions. The transfected cells which expressed target gene were confirmed by quantitative RT-PCR.

### Cell proliferation assay

Cell proliferation assays were performed with Cell Counting Kit-8 (CCK-8, Dojin, Japan) according to the manufacturer's instructions. Cell growth was monitored every 24 h over 5 days, and for each time point was carried out in duplicate. The absorbance was measured for each well at a wavelength of 450 nm.

### Wound healing assay

For the wound healing assay, the human gastric cancer cell lines, BGC-823 and SGC-7901 (1×10^5^ cells/35×11 mm dishes) were seeded and incubated for 24 hours at 37°C and then transfected with recombinant plasmids. After achieving confluence, the cellular layer in each plate was scratched using a plastic pipette tip. The migration of the cells at the edge of the scratch was analyzed at 0, 24 and 48 hours, the images were captured and analyzed by Leica fluorescence microscope and matched image analysis software (Comet Assay IV Image analysis system, PI, UK).

### Routine Tumor Maker Detection

Clinical and biochemical data from the patients are summarized in Table 1. Routine biochemical tests were measured using standard methods and matched reagents (Hitachi 7600 Analyzer (Hitachi, Tokyo, Japan). Tumor marker levels, including carbohydrate antigen 19-9 (CA19-9), carcino-embryonic antigen (CEA), Cytokeratin 19 (CK19), CA125 and CA724, were determined on a Roche E170 modular with matched reagents.

### Statistical Analysis

All quantitative variables were expressed as means±standard deviations unless stated otherwise. Quantitative variables were compared with Student t tests, analyses of variance (ANOVA), or nonparametric tests. Pearson coefficients of correlation (Spearman coefficients of correlation were calculated for ordinal categorical variables) and their associated probabilities (*p*) were used to evaluate correlations between parameters. All reported *p* values were 2-tailed, and *p* values<0.05 were considered statistically significant. Statistical analyses were performed with SPSS 16.0 for Windows statistical software (SPSS Inc.).

## Results

### Different serum N-Glycan profiling patterns in gastric cancer, gastric ulcer and healthy controls

**Figure 1:**
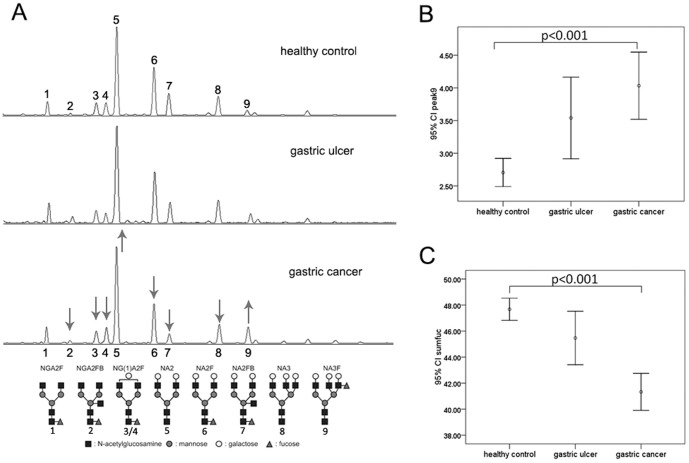
Typical desialylated N-glycan profiles from total serum protein and N-glycan values differ significantly among healthy control, gastric ulcer and gastric cancers. (A) The three panels (from top to bottom) are typical serum N-glycan profiles from healthy control, gastric ulcer and gastric cancers. Nine major peaks can be identified. The structures of the N-glycan peaks are shown below the chart. Levels in peaks 5 and 9 are elevated (up arrows), and levels in peaks 2, 3, 4, 6, 7 and 8 are decreased (down arrows) in gastric cancer compared with that in healthy and disease controls. Peaks 1, 2, 3, 4, 6 and 7 are core-fucosylated glycans. (B) The value of peak9 (branching α-1,3-fucosylated triantennary N-glycan, NA3FB) is elevated sequentially from healthy control, gastric ulcer to gastric cancer (P<0.001), Error bars represent 95% confidence intervals (95%CI) for means. (C) The value of sumfuc is decreased in gastric cancer (P<0.001). Sumfuc indicates the total abundance of core-fucosylated structure glycans (peaks 1, 2, 3, 4, 6 and 7). Error bars represent 95%CI for means.

Using DSA-FACE, we examined the N-glycan profiles in desialylated sera from patients with gastric cancer (n = 80), gastric ulcer (n = 25), and age matched healthy individuals (n = 139). We quantified and statistically compared these peaks among 3 different groups. At least 9 N-glycan structures (peaks) were identified in all samples (Figure 1A). The structural analysis of these N-glycans was published previously by Callewaert [11]. The average relative abundance of these N-glycan structures was described in Table 2. The abundance of structures in peaks 2, 3, 5, 6, 7, 8 and 9 revealed statistically significant differences among the gastric cancer, gastric ulcer, and healthy control groups, indicating that different N-glycan patterns appeared in different pathophysiologic conditions. Compared with the healthy control group, peak5 (bigalacto biantennary glycan, NA2) and peak9 (branching α-1,3-fucosylated triantennary glycan, NA3Fb) were elevated (P<0.001)(Figure 1B); whereas core- fucosylated peaks, such as peak2 (agalacto core-α-1,6-fucosylated bisecting biantennary glycan, NGA2FB), peak3(single agalacto core-α-1,6-fucosylated biantennary glycan, NG1A2F), peak6 (bigalacto core-α-1,6-fucosylated biantennary glycan, NA2F) and peak7(bigalacto core-α-1,6-fucosylated bisecting biantennary glycan, NA2FB) were decreased in the gastric cancer group (P<0.001). Similarly, the abundance of core-fucosylated structure glycans named sumfuc (indicates total core-fucosylated structures, including peak 1, 2, 3, 4, 6, and 7) was decreased significantly in the gastric cancer (P<0.001) (Figure 1C). The gastric cancer group was further classified into 4 subgroups according to TNM Staging of UICC for gastric cancer. The structure abundance and sumfuc did not change significantly among the 4 different subgroups; however, sumfuc was decreased gradually with progression stages of gastric cancer (Table 3).

**Table 2:** General N-glycome profiling results in 3 different groups.

	healthy control	gastric ulcer	gastric cancer	oneway ANOVA	
	(means±SD, n = 139)	(means±SD, n = 25)	(means±SD, n = 80)	F value	*p* value
age	54.82±5.70	54.52±7.33	54.35±6.69	0.16	0.857
peak1	7.54±1.99	7.20±2.81	6.97±2.74	1.53	0.22
peak2	1.23±0.37	1.05±0.38	0.99±0.36	11.52	<0.001
peak3	6.42±1.24	5.98±1.21	5.24±1.13	24.08	<0.001
peak4	5.64±1.04	5.34±0.86	5.72±0.82	1.53	0.219
peak5	41.35±3.85	43.76±3.66	46.94±4.95	44.53	<0.001
peak6	20.67±2.63	19.60±2.43	17.17±3.44	36.94	<0.001
peak7	6.15±1.46	6.28±0.99	5.21±1.15	14	<0.001
peak8	8.26±1.93	7.23±1.94	7.69±2.30	3.78	0.024
peak9	2.70±1.28	3.54±1.51	4.03±2.31	15.78	<0.001
sumfuc	47.67±5.08	45.46±4.98	41.32±6.39	33.37	<0.001

sumfuc = peak1+peak2+peak3+peak4+peak6+peak7.

**Table 3:** N-glycome analysis in gastric cancer patients at different progression stages.

	TNM1	TNM2	TNM3	TNM4	oneway ANOVA	
	(means±SD, n = 13)	(means±SD, n = 24)	(means±SD, n = 30)	(means±SD, n = 13)	F value	p value
peak1	7.13±2.31	6.91±2.72	7.95±3.17	7.18±3.17	0.82	0.488
peak2	1.04±0.33	0.97±0.33	1.13±0.39	0.93±0.38	1.84	0.145
peak3	6.20±1.00	5.61±1.37	5.62±1.36	5.53±1.27	0.99	0.403
peak4	6.00±0.74	5.59±0.83	5.91±1.09	5.25±0.69	3.13	0.029
peak5	43.80±3.37	45.95±4.39	45.55±4.82	45.68±4.77	0.83	0.479
peak6	19.43±3.14	18.53±2.61	17.15±3.24	18.10±2.81	2.66	0.052
peak7	5.79±0.89	5.81±1.14	5.36±1.35	5.41±1.33	1.00	0.398
peak8	7.37±1.86	7.19±2.01	7.18±2.81	7.63±2.52	0.19	0.906
peak9	3.20±1.15	3.42±1.62	4.12±1.94	4.25±2.16	1.82	0.149
sumfuc	45.61±4.52	43.43±5.74	43.13±6.72	42.42±6.44	0.89	0.448
CA19-9	15.94±38.30	18.05±38.07	57.27±126.86	47.55±117.58	1.20	0.312
CEA	1.63±0.90	7.18±15.06	4.91±8.77	8.94±15.76	1.40	0.249
CK19	0.66±0.54	0.90±0.71	1.83±3.24	10.76±24.54	4.14	0.008
CA125	4.70±3.81	5.30±4.27	10.35±19.09	42.79±114.26	2.61	0.055
CA724	0.71±0.38	1.03±1.13	2.25±3.72	13.84±30.13	4.65	0.004

### Correlation between N-glycans and routine tumor markers

To date, CEA is still used widely for the screening and monitoring of gastric cancer. We analyzed the correlations among individual N-glycan marker with CA19-9, CEA, CK19, CA125 and CA724. The Pearson correlation analysis indicated that CEA was associated positively with peak1 (r = 0.19; P<0.01), and peak9 (r = 0.28; P<0.01), whereas negatively with peak6 (r = −0.18; P<0.01) and peak8 (r = −0.23; P<0.01) (Table 4).

**Table 4:** Correlation analysis between N-glycans and routine tumor markers of gastric cancer.

Correlations		CA199	CEA	CK19	CA125	CA724
peak1	r	0.02	0.19	0.01	0.03	0.01
	p	0.83	<0.01	0.9	0.76	0.97
peak2	r	−0.03	0.05	−0.13	−0.1	−0.12
	p	0.68	0.45	0.12	0.22	0.16
peak3	r	−0.05	−0.05	−0.07	−0.03	−0.05
	p	0.57	0.48	0.44	0.74	0.58
peak4	r	0.09	−0.02	−0.01	0.06	0.04
	p	0.25	0.82	0.91	0.5	0.62
peak5	r	0.08	0.05	0.07	0.03	0.06
	p	0.33	0.43	0.44	0.71	0.48
peak6	r	−0.04	−0.18	−0.02	−0.06	−0.04
	p	0.61	0.01	0.78	0.47	0.64
peak7	r	−0.1	−0.07	−0.17	−0.05	−0.14
	p	0.22	0.3	0.04	0.58	0.11
peak8	r	−0.23	−0.23	−0.07	−0.07	−0.1
	p	<0.01	<0.01	0.42	0.42	0.26
peak9	r	0.21	0.28	0.15	0.12	0.18
	p	0.01	<0.01	0.07	0.15	0.04
Sumfuc	r	−0.03	−0.03	−0.07	−0.03	−0.06
	p	0.7	0.63	0.44	0.71	0.5

### Comparison of sumfuc between gastric cancer and other digestive system cancers

Previously we have studied the N-Glycan profiling of hepatocellular carcinoma (HCC) [19] and colorectal cancer (CRC) [20]. The abundance of sumfuc revealed statistically significant differences among the gastric cancer (41.32±6.39), HCC (50.99±8.39), CRC (45.33±5.96) and healthy control (47.67±5.08) groups (P<0.001, Table 5), indicating that different level of core-fucosylation appeared in cancers with different tissue origins. Among the 4 groups, the level of core-fucosylation in HCC was the highest, while the fucosylation level in gastric cancer was the lowest, and was even lower than that in healthy control.

**Table 5:** N-glycome analysis in digestive system cancers

	Healthy control	HCC	CRC	GC	p value
	(means±SD, n = 139)	(means±SD, n = 100)	(means±SD, n = 100)	(means±SD, n = 80)	
age	54.82±5.70	53.64±10.66	54.41±6.67	54.35±6.69	0.863
sumfuc	47.67±5.08	50.99±8.39	45.33±5.96	41.32±6.39	<0.001

### N-Glycan profiling patterns in gastric tumor and non-tumor tissues

**Figure 2:**
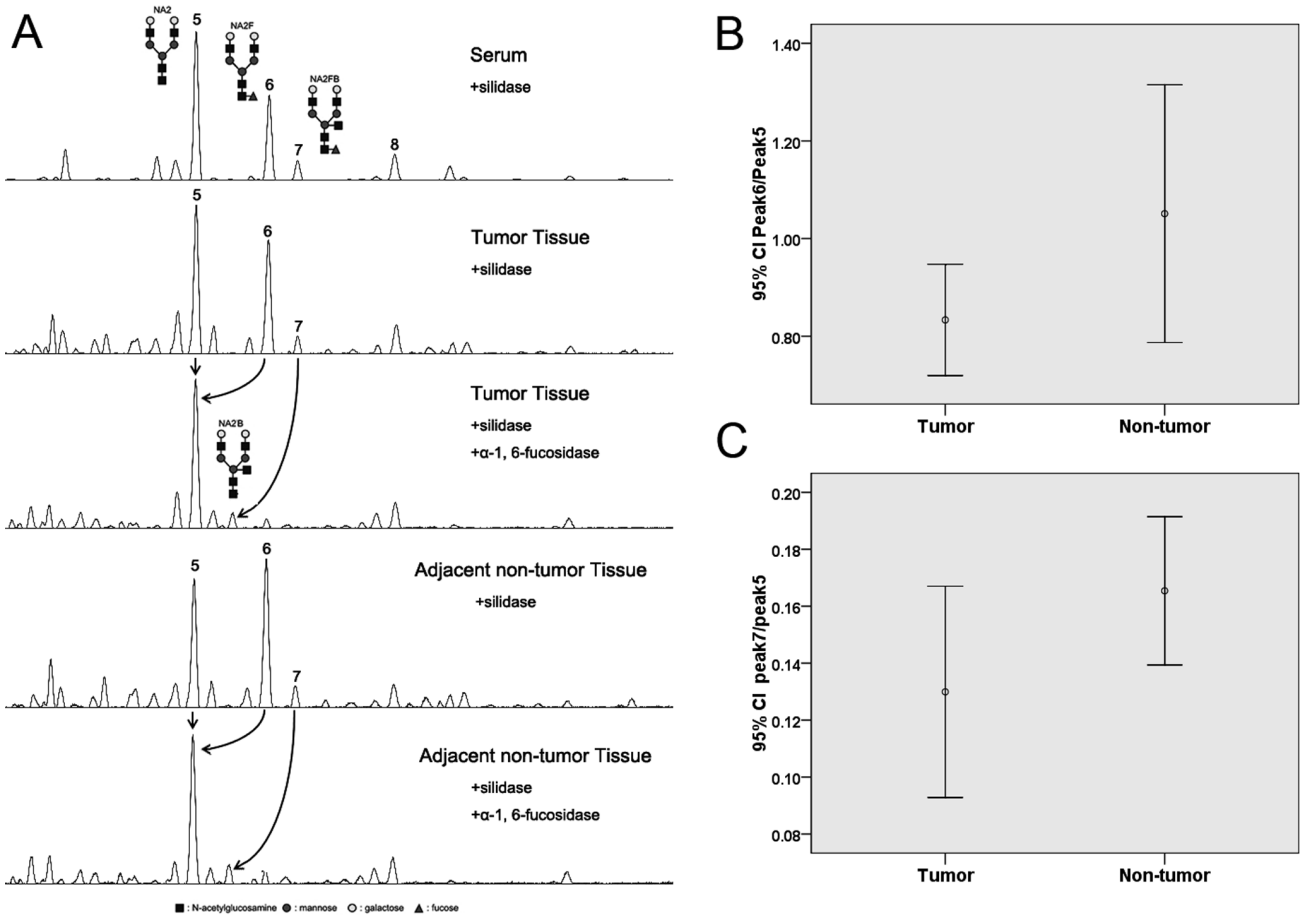
Typical desialylated and α-1,6-fucosidase digested N-glycan profiles from gastric tumor and adjacent non-tumor tissue proteins. (A) The five panels (from top to bottom) are desialylated N-glycan profiles from serum as reference, desialylated N-glycan profiles from tumor tissues, desialylated N-glycan profiles from tumor tissues treated with α-1,6-fucosidase digestion, desialylated N-glycan profiles from adjacent non-tumor tissues, and desialylated N-glycan profiles from adjacent non-tumor tissues treated with α-1,6-fucosidase digestion. The arrow lines indicate the changes of N-glycan peaks treated with α-1,6-fucosidase digestion. Peak6 and peak7 remove forward after α-1,6-fucosidase digestion, which reveale that peak5, 6 and 7 have the same structures as in the serum, and peak6 and peak7 are α-1,6-fucosylated N-glycans. (B) The value of peak6/peak5 is decreased in tumor tissues, but the difference is not statistically significant. Error bars represent 95%CI for means. (C) The value of peak7/peak5 is also decreased in tumor tissues, but the difference is not statistically significant.

N-glycan profiles were examined in desialylated protein from gastric tumor and non-tumor tissues. The three most abundant bigalacto biantennary glycans showed in serum, peak5(NA2), peak6(NA2F) and preak7(NA2FB) were identified also in tissue proteins (Figure 2A). Peak6 and Peak7 were confirmed to be core-fucosylated structures dervied from peak5 after treated with core-α-1, 6-fucosidase digestion (Figure 2A). The heights of these three peaks were analyzed using GeneMapper software. The ratio of peak6 to peak5 was lower in tumor tissues than that in adjacent non-tumor tissues (0.83±0.18 vs. 1.05±0.42, Figure 2B), as well as the ratio of peak7 to peak5 (0.13±0.06 vs. 0.16±0.04, Figure 2C). However, there were no statistically significant differences (P>0.05) between tumor and non-tumor tissues.

### Decreased levels of total core fucosylation identified in both sera and tissues from gastric cancer

**Figure 3:**
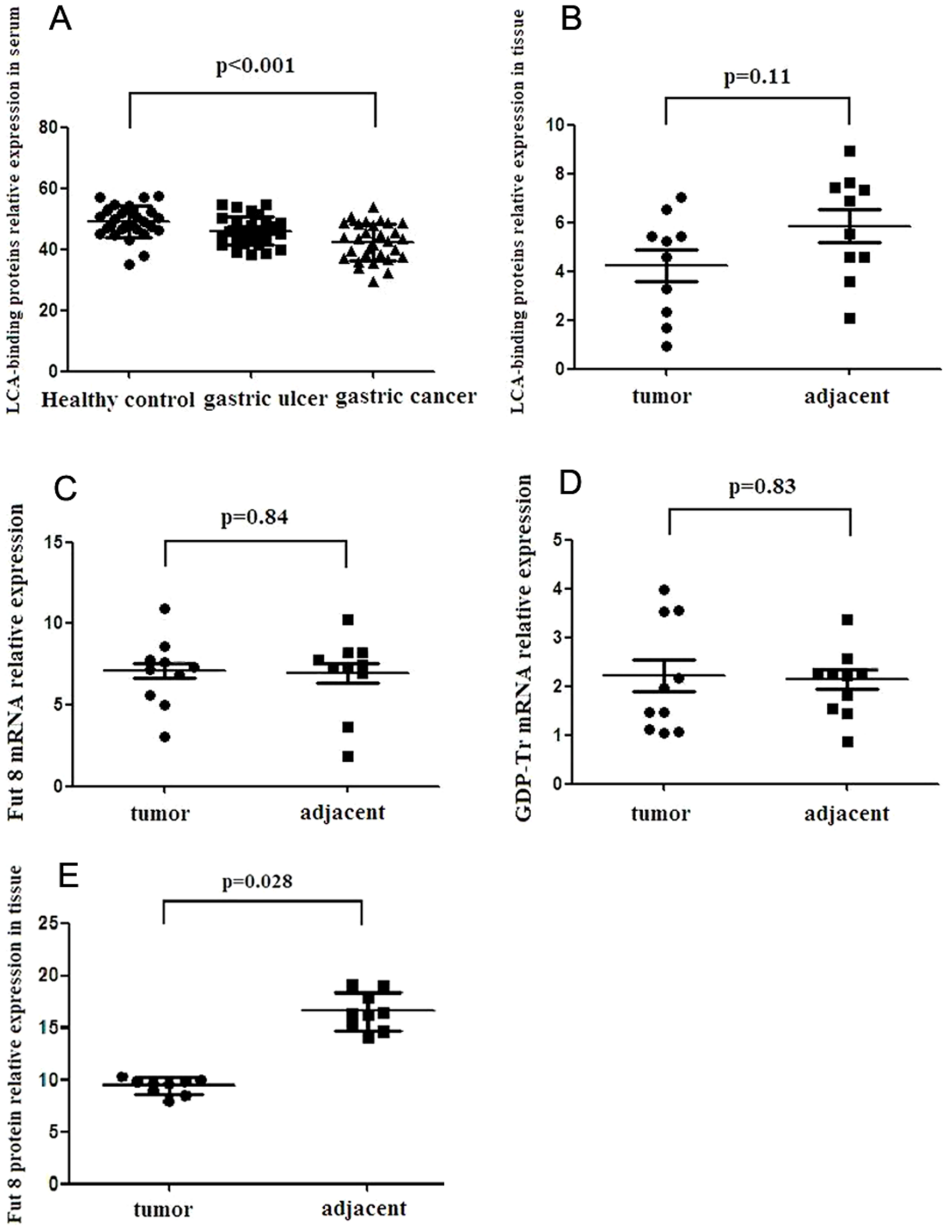
The abundance of total core-fucosylated residues from lens culinaris agglutinin A (LCA) lectin blot, mRNA level of α-1,6-fucosyltransferase (Fut8), guanosine diphosphate (GDP)-fucose transporter (GDP-Tr) from quantitative RT-PCR, and protein level of Fut8 from western blot. (A) Lectin blots of serum proteins were probed with LCA. The horizontal axis represents the experimental groups: healthy control (n = 30), gastric ulcer (n = 25), and gastric cancer (GC) (n = 30); the vertical axis indicates the ratio of fucosylated proteins to total proteins. The level of fucosylated proteins in gastric cancer is the lowest among 3 groups (P<0.001). (B) Lectin blots from tissue proteins were probed with LCA. The horizontal axis represents the experimental groups: tumor tissues (n = 10) and adjacent tissues (n = 10). The vertical axis indicates the ratio of fucosylated proteins to total proteins. The core-fucosylated level was lower in gastric tumor, but the difference showed no statistical significance (P>0.05). (C) and (D) Relative messenger RNA (mRNA) expression levels of Fut8 or GDP-Tr in tissues were measured by quantitative RT-PCR. The horizontal axis represents the experimental groups: tumor tissues (n = 10) and adjacent tissues (n = 10). The vertical axis indicates the relative expression levels of Fut8 or GDP-Tr. The difference between groups was not statistically significant (P>0.05). (E) The relative abundance of Fut8 protein was illustrated using western blot. The horizontal axis represents the experimental groups: tumor tissues (n = 9) and adjacent tissues (n = 9). The vertical axis indicates the relative levels of Fut8 protein. The relative abundance of Fut8 protein in adjacent tissues was significantly higher than that in tumor tissues (P<0.05).

**Figure 4:**
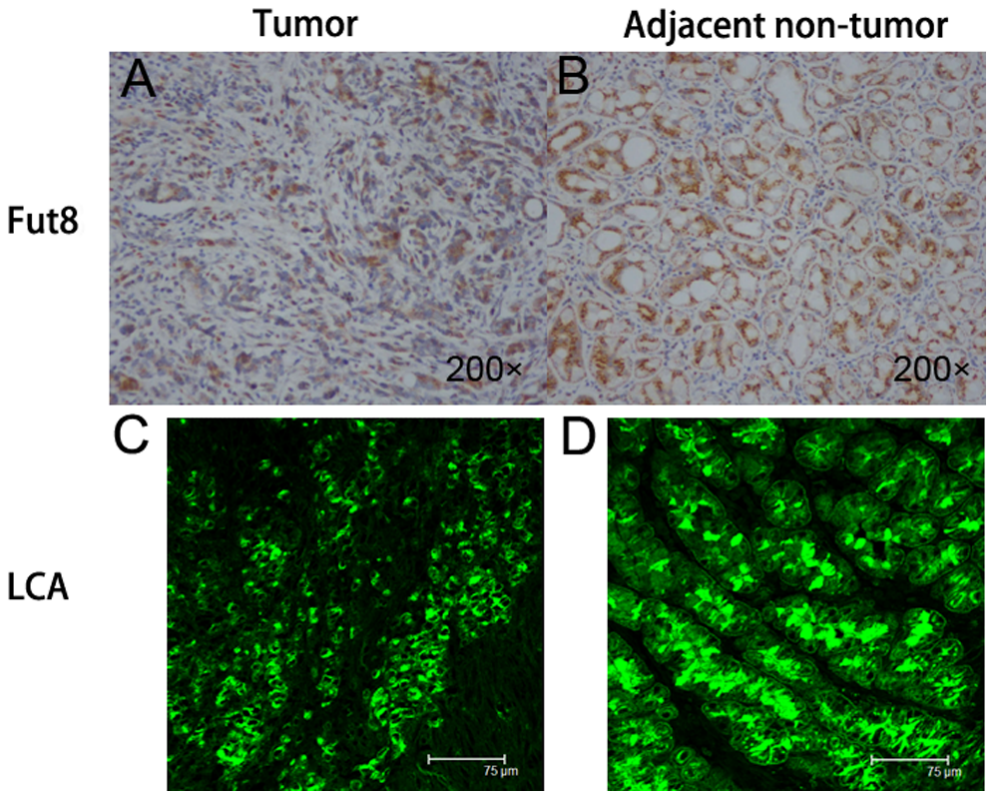
Immunohistochemical staining with Fut8 and lectin-histochemical staining with LCA in typical gastric tumor tissues and adjacent non-tumor tissues. (A) Immunohistochemical staining with Fut8 in tumor cells (200×), the expression of Fut8 is weakly positive. (B) Immunohistochemical staining with Fut8 in non-tumor gastric cells (200×), the expression of Fut8 is strongly positive. (C) Lectin-histochemical staining with LCA in tumor cells, bar 75 µm, the expression of LCA-binding core-fucosylated proteins is weakly positive. (D) Lectin-histochemical staining with LCA in non-tumor gastric cells, bar 75 µm, the expression of core-fucosylated proteins is strongly positive.

Since LCA can specifically recognize the glycoproteins with α-1,6-fucosylated-linked N-acetyl-D-glucosamine-asparagine (GlcNAc-Asp) in the trimannosyl core, we investigated the total core fucosylated proteins from sera and tissues in gastric cancer using LCA to validate the finding in DSA-FACE. In serum, the level of LCA-binding core fucose residues was lower in gastric cancer than that in the gastric ulcer and healthy controls (Figure 3A). Total core fucose abundance also trended to be lower in gastric tumors compared to that in paired adjacent tissues (Figure 3B). To determine whether the alteration of total core fucosylation in gastric tumor tissue is relevant to alteration of the glycosylation biosynthesis pathway, quantitative RT-PCR was used to analyze the abundance of mammalian Fut8 and GDP-Tr in gastric tumors and adjacent tissues. The results revealed that no significant difference of Fut8 and GDP-Tr mRNA expression were observed between tumors and adjacent tissues (Figure 3C and 3D). The relative abundance of Fut8 protein was illustrated using western blot (raw data was shown in Figure S1). The Fut8 in adjacent tissues was significantly higher than that in tumor tissues (P<0.05) (Figure 3E). Immunohistochemistry revealed that the Fut8 was strongly positively expressed at the luminal borders of non-tumor gastric cells (Figure 4B), however it weakly positively expressed in tumor cells (Figure 4A). The expression of LCA-binding core-fucosylated glycoproteins was higher in non-tumor gastric cells than that in tumor cells (Figure 4C, 4D). So, the level of total core fucosylation was identified decreased in both sera and tissues from gastric cancer.

### Effect of Fut8 and GDP-Tr on proliferation and migration in human gastric cancer cell lines

**Figure 5:**
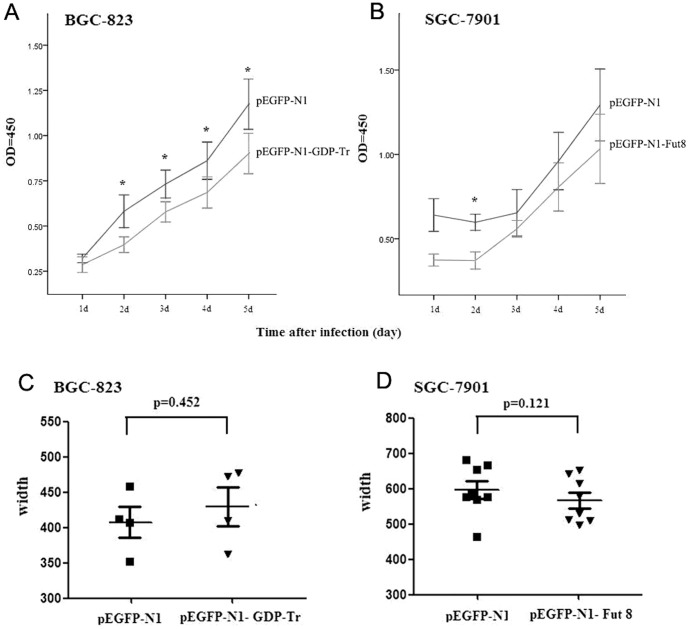
The proliferation and wound healing migration assay of gastric cancer cells transfected with recombinant expression plasmids. (A) and (B) The proliferation of BGC-823 cells and SGC-7901 cells after transfection with pEGFP-N1-GDP-Tr and pEGFP-N1-Fut8 respectively was analyzed by Cell Counting Kit-8 (CCK-8) assays. Representative results from four independent experiments are shown. * P<0.05 compared with control group. (C) and (D) The wound healing migration assay of BGC-823 cells after transfection with pEGFP-N1-GDP-Tr as well as SGC-7901 cells after transfection with pEGFP-N1-Fut8. The gap distance (µm) in migration assay can be quantitatively evaluated using softwares. Representative results from 3 independent experiments are shown. P>0.05 compared with control group.

The human gastric cancer cell lines, BGC-823 and SGC-7901 were used in vitro study. Since BGC-823 expressed lower level of GDP-Tr while SGC-7901 expressed lower level of Fut8 in our pilot research, pEGFP-N1-GDP-Tr was transfected into BGC-823 for overexpressing GDP-Tr and pEGFP-N1- Fut8 was transfected into SGC-7901 for overexpressing Fut8. Recombinant expressions were successfully achieved shown by reporter gene GFP expression (Figure S2). To examine the possible involvement of GDP-Tr and Fut8 in tumor proliferation and migration, BGC-823 and SGC-7901 cells were investigated after transfection using CCK-8 and wound healing migration assay respectively. The results showed that upregulation of GDP-Tr decreased BGC-823 proliferation at 2-5 days after transfection and upregulation of Fut8 decreased SGC-7901 proliferation at 2 days after transfection (Figure 5A and 5B). These results indicated that high expression of GDP-Tr and Fut8 could suppress the proliferation of human gastric cancer cells. Wound healing assay demonstrated that GDP-Tr and Fut8 have no significant influence on migration in BGC-823 and SGC-7901 at 48 h after treatment (Figure 5C and 5D).

## Discussion

Glycosylation is one of the most common post-translational modifications appeared in about 70% of all known proteins. Alterations in glycosylation play a role in a diverse set of biological phenomena such as tumor cell metastasis, intracellular communication and inflammation [21]-[23]. More and more studies indicate that the alterations of glycosylation and the levels of glycosyltransferases activities derived from the malignant transformation are relevant to human malignancies [24]–[25]. DSA-FACE is a simple and efficient technology for measuring N-glycan changes in serum. We previously used this technology to assist in the diagnosis of HCC and CRC [19]–[20]. In the current study, we used it to analyze characteristic N-linked profiling pattern in gastric cancer. The results indicated that there were significantly different N-Glycan profiling patterns among gastric cancer, gastric ulcer and healthy controls. Among these different profiling patterns, peak9 and sumfuc were found to be changed in an opposite pattern in gastric cancer compared to those in controls. As shown by previous research, peak9 was associated with the development of varieties of malignancies, we confirmed again that the triantennary N-glycan structure abundance increased significantly in gastric cancer and this change had been revealed to be correlated with CEA, which is a most commonly used CRC marker and is a highly heterogeneous glycoprotein that contains 60% carbohydrate [26]. In individual N-glycan structure abundance analysis, we observed that sumfuc was decreased significantly in gastric cancer compared with controls. For further validate this finding, we performed lectin blot in both sera and tissues. By using HCC serum as a positive control, we observed decreased fucosylation of N-glycans on total proteins in sera from gastric cancer. A similar trend was revealed in gastric tumor tissues. These findings are consistent with our previous reports on CRC [20]. However the results are contrary to previous reports on cancers with other tissue origins. The increases in fucosylated oligosaccharides under pathological conditions have been reported by others and our group [19]. The fucosylated AFP (AFP-L3) has been used as HCC marker in clinical practice [25]. The contradictories of the core-fucosylation levels appeared in different cancers might be due to the different roles played by different tissues, e.g. liver is the major organ responsible for producing glycoproteins besides immunoglobulin-producing B-lymphocytes [27]–[28].

Fucosylation is catalyzed by fucosyltransferases, guanosine 5′-diphosphate (GDP)-fucose synthetic enzymes, and GDP-fucose transporter(s). GDP-fucose is a common donor substrate to all fucosyltransferases. After GDP-fucose has been synthesized in the cytosol, it is transported to the Golgi apparatus through GDP-Tr to serve as a substrate for fucosyltransferases [17], [29]. Therefore, GDP-Tr is a key factor for the GDP-fucose synthesized pathway. Fut8 is the only fucosyltransferase involved in core-fucosylation [29]. We detected decreased Fut8 protein expression in tumor tissue, which supported our findings in sera. However, Fut8 mRNA expression was no significant difference in gastric tumors and adjacent tissues. Glycosylation is highly reflective of changes in the environment of a cell. Epigenetic modifications to the genome are stably transmitted to daughter cells without the requirement for genetic sequence alterations.[30] To clarify whether the decreased core fucosylation plays an important role in carcinogenesis of gastric cancer, we extended the study in gastric cell lines in vitro. We found that the up-regulated GDP-Tr and Fut8 expression in human gastric cancer cells could lead to low proliferation. But we failed to find any impact of core-fucosylation on cell migration. These results help to explain the potential mechanism why decreased core-fucosylation appeared in gastric cancer and this down-regulated core-fucosylation correlated with some malignant biological behavior of gastric cancer cells.

In the recent years, there are several findings revealing that the alterations of glycosylation play important roles in progression of diseases besides liver cancer and autoimmune diseases. The significant alterations in the glycosylation of secreted glycoproteins included a reduction in core fucosylation, increased branching and increased sialylation, and modifications to the epigenetic machinery have a profound effect on the glycan structures generated by cells during ovarian cancer progression[30]. The levels of core-fucosylated biantennary glycans and α-2,3-linked sialic acids were significantly increased in prostate cancer.[31] There was an increase of 40% in core fucosylation in the main sialylated biantennary glycans in the pancreatic cancer serum, which would be indicative of a subset of tumor-associated glycoforms[32].

Recently, the function of core-fucosylation was reported to be associated with the function of EGFR [33]. The binding of EGF to its receptor requires core fucosylation of N-glycans of EGFR. Decreased core-fucosylation in gastric cancer might be associated with lower core-fucosylation of EGFR, and thus with reduced activation of EGF-induced phosphorylation of the EGFR pathway. Up to now, the mechanism behind the alteration of fucosylation during gastric cancer development is not fully understood.

As to the source of abnormal glycosyaltion found in sera, increasing evidences have shown that abnormal glycosylation do appear in circulation in addition to diseases of liver and B lymphocytes[20.27.34]. Our study and the others revealed that malignant tissues at least partially contributed to glycosylation alterations in circulation[20.35].In addition, immunoglobulins, the major glycoproteins in circulation were found to be secreted from some tumor tissues and tumor cell lines [36]–[37]. The malignant related IgG showed special glycosylation in structure and stimulated cell proliferation in a pattern similar to growth factors functionally [38]. In brief, the exact mechanisms on the alteration of peripheral N-glycome in gastric cancer require further exploration to elucidate.

There are some limitations of this study. First, LCA was used for core-fucosylation abundance analysis by lectin blot in this study. However LCA binds not only to fucose but also to mannose residues in N-glycans. Recently PhoSL, a lectin binds only to core α-1,6-fucosylated oligosaccharides reported by a Japanese group in 2012, shows more specific than LCA in core-fucosylation assessment[39]. Unfortunately, the availability of PhoSL is limited and its feasibility in clinical application in limited. LCA is now still an alternative lectin widely used for detecting α-1,6-fucosyl-linked sugar chains.Second, although both tissue and serum studies revealed similar finding indicating the low core-fucosylation in gastric cancer, whether the alteration of N-glycomic core-fucosylation was caused by tumor or tumor microenvironment was not fully addressed in this study. In vivo animal model study is required in future to elucidate the mechanism how peripheral glycosylation is regulated in non-typical glycosylation tissues malignancies. Third, the functional implication of low fucosyation in gastric cancer is not well investigated. The N-glycan structural alteration of some important receptors might modulate the signal transduction pathway. Future extended clinical study and functional exploration are required to validate the finding revealed in this study and uncover some other yet unknown mechanism involved to elucidate the impacts of glycosyations in carcinogenesis.

## Supporting Information

Figure S1 **Raw figures of Fut8 Western blotting in tumor and adjacent tissue.** Western blot analysis showed that the Fut8 in adjacent tissues was significantly higher than that in tumor tissues. T indicates tumor tissue, N indicates adjacent tissue, M indicates protein marker. NC indicates Purified albumin as negative control. Raw figures of Fut8 Western blotting (A) from 1th to 4th matched pairs, (B) from 5th to 7th matched pairs, (C) from 8th to 9th matched pairs.
(JPG)

Figure S2 **The Images under microscope of transfection efficiency of gastric cancer cells.** A: The image of BGC-823 cells after transfection with pEGFP-N1-GDP-Tr taken under fluoroscope light. B: The image of BGC-823 cells after transfection with pEGFP-N1-GDP-Tr taken under natural light. C: The image of SGC-7901 cells after transfection with pEGFP-N1-Fut8 taken under fluoroscope light. D: The image of SGC-7901 cells after transfection with pEGFP-N1-Fut8 taken under natural light.
(JPG)

Table S1 PCR primer pairs used in quantitative RT-PCR.
(DOCX)

Table S2 PCR primer pairs used in Recombinant Plasmids Construction.
(DOCX)
