# Supplementary figures and images for: Decreased Core-Fucosylation Contributes to Malignancy in Gastric Cancer
Source: PLoS One. 2014 Apr 14;9(4):e94536. doi: 10.1371/journal.pone.0094536 (PMC3986093; doi:10.1371/journal.pone.0094536)

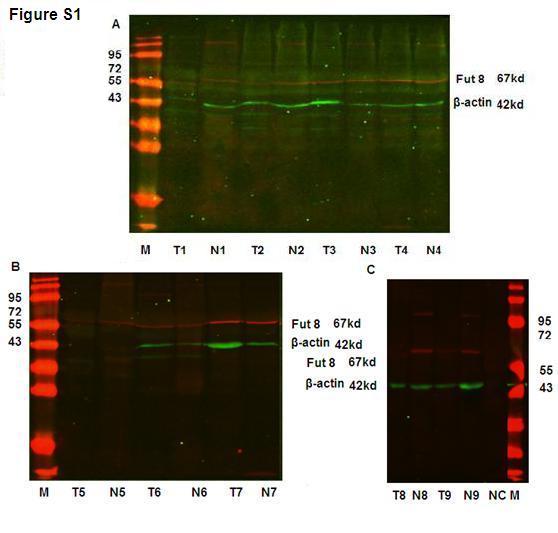

Supplement: Figure S1 — Raw figures of Fut8 Western blotting in tumor and adjacent tissue. Western blot analysis showed that the Fut8 in adjacent tissues was significantly higher than that in tumor tissues. T indicates tumor tissue, N indicates adjacent tissue, M indicates protein marker. NC indicates Purified albumin as negative control. Raw figures of Fut8 Western blotting (A) from 1th to 4th matched pairs, (B) from 5th to 7th matched pairs, (C) from 8th to 9th matched pairs. (JPG) [file pone.0094536.s001.jpg]

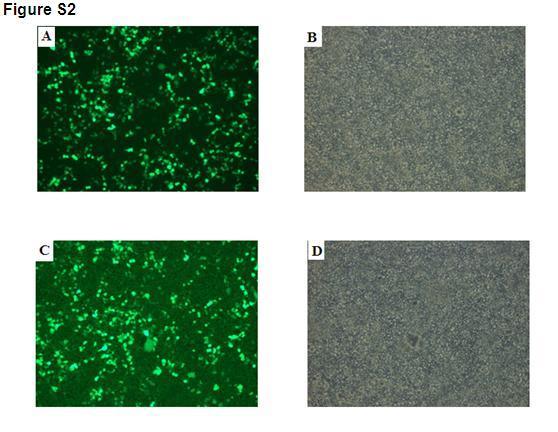

Supplement: Figure S2 — The Images under microscope of transfection efficiency of gastric cancer cells. A: The image of BGC-823 cells after transfection with pEGFP-N1-GDP-Tr taken under fluoroscope light. B: The image of BGC-823 cells after transfection with pEGFP-N1-GDP-Tr taken under natural light. C: The image of SGC-7901 cells after transfection with pEGFP-N1-Fut8 taken under fluoroscope light. D: The image of SGC-7901 cells after transfection with pEGFP-N1-Fut8 taken under natural light. (JPG) [file pone.0094536.s002.jpg]
