# Supplementary material for: Decreased Core-Fucosylation Contributes to Malignancy in Gastric Cancer
Source: PLoS One. 2014 Apr 14;9(4):e94536. doi: 10.1371/journal.pone.0094536 (PMC3986093; doi:10.1371/journal.pone.0094536)
Supplement: Table S1 — PCR primer pairs used in quantitative RT-PCR. (DOCX) [file pone.0094536.s003.docx]

Table S1. PCR primer pairs used in quantitative RT-PCR

| Gene | Forward Primer (5’-3’) | Reverse Primer (5’-3’) |
| --- | --- | --- |
| Fut8 | 5'CCTGGCGTTGGATTATGCTCA 3' | 5'CCCTGATCAATAGGGCCTTCT 3' |
| GDP-Tr | 5'CTGCCTCAAGTACGTCGGTG 3' | 5'CCGATGATGATACCGCAGGTG 3' |
| β-actin | 5'TGTTGCCATCAATGACCCCTT 3' | 5'CTCCACGACGTACTCAGCG 3' |
| GAPDH | 5'AGGGCTGCTTTTAACTCTGGT 3' | 5'CCCCACTTGATTTTGGAGGGA 3' |
