# Supplementary material for: Decreased Core-Fucosylation Contributes to Malignancy in Gastric Cancer
Source: PLoS One. 2014 Apr 14;9(4):e94536. doi: 10.1371/journal.pone.0094536 (PMC3986093; doi:10.1371/journal.pone.0094536)
Supplement: Table S2 — PCR primer pairs used in Recombinant Plasmids Construction. (DOCX) [file pone.0094536.s004.docx]

Table S2. PCR primer pairs used in Recombinant Plasmids Construction

| Gene | Forward Primer (5’-3’) | Reverse Primer (5’-3’) |
| --- | --- | --- |
| Fut8 | 5' CCCTCGAGATGCGGCCATGGACTGGTTC 3' | 5' GGGGTACC TTTCTCAGCCTCAGGATATGTG 3' |
| GDP-Tr | 5' CCCTCGAG ATGAATAGGGCCCCTCTGAAG 3'' | 5' GGGGTACC CACCCCCATGGCGCTCTTC 3' |
